# Cerebrovascular pathology and neurovascular coupling impairment in aged‐mouse model of Alzheimer’s disease

**DOI:** 10.1002/alz.095727

**Published:** 2025-01-09

**Authors:** Moltira Promkan, Kamonchat Phikulthong, Rungruedee Kimseng, Kate Pauss, Tiffany Lee, Erica M. Weekman, Peter T Nelson, Donna M. Wilcock, Pradoldej Sompol

**Affiliations:** ^1^ University of Kentucky, Lexington, KY USA; ^2^ Sanders‐Brown Center on Aging, Lexington, KY USA; ^3^ Indiana University School of Medicine/Stark Neurosciences Research Institute, Indianapolis, IN USA; ^4^ University of Kentucky College of Medicine, Sanders‐Brown Center on Aging, Lexington, KY USA; ^5^ Indiana University School of Medicine, Stark Neurosciences Research Institute, Department of Neurology, Indianapolis, IN USA

## Abstract

**Background:**

Vascular pathology profoundly comorbid with AD pathology could worsen disease progression and reduce treatment efficacy. Knowledge of small vessels and cerebrovascular function in AD mouse models is limited. Investigating vascular related aspects for preclinical AD studies is essential for biomarker development and treatment trials. Therefore, we aim to characterize cerebrovascular amyloid angiopathy (CAA), vascular degeneration, and cerebrovascular function in an aged Tg2576 mouse model of AD.

**Method:**

WT and Tg2576 (∼ 2 years of age) were housed in a reversed light cycle room. Cranial window surgery and cranial window installation were performed. After 3 weeks of recovery, the animals were acclimated to an intravital multiphoton imaging platform. To visualize beta‐amyloid in the brain, Methoxy‐X04 (10mg/kg) was injected the day before the imaging. Cerebrovasculature was visualized by intravascular retro‐orbital injection of rhodamine‐dextran (5% V/W in saline). This procedure was done while the animals were under anesthesia and securely head‐fixed prior to the imaging. Z‐stack imaging was performed, and vascular structure was analyzed by using FIJI or ImageJ. Neurovascular coupling was performed to investigate vascular function in awake mice. While imaging penetrating arteriole, air‐puff stimulation of contralateral whiskers was conducted and increased vascular diameter is used as an indicator of hyperemic neurovascular function.

**Result:**

Investigation of cerebrovascular pathology including CAA, vascular straightness, and vascular blebbing are ongoing. During whisker stimulation, vascular diameter was relatively reduced in Tg2576 compared to WT control mice.

**Conclusion:**

Aged Tg2576 exhibits comorbidity of amyloid plaques, cerebral amyloid angiopathy, small vessel pathology and cerebrovascular dysfunction similar to human brain. This aged Tg2576 could be used as a preclinical translational mixed vascular/AD model.

